# Complete mitochondrial genome of *Xiphidiopsis* (*Xiphidiopsis*) *gurneyi* (Orthoptera, Tettigoniidae, Meconematinae)

**DOI:** 10.1080/23802359.2018.1476073

**Published:** 2018-05-21

**Authors:** Shao Li Mao, Zhong Ying Qiu, Qian Li, Yang Li, Ya Fu Zhou

**Affiliations:** a Xi’an Botanical Garden of Shaanxi Province/Institute of Botany of Shaanxi Province, Xi’an, China;; b Shaanxi Key Laboratory of Brain Disorders & School of Basic Medical Sciences, Xi’an Medical University, Xi’an, China

**Keywords:** Meconematinae, *Xiphidiopsis* (*Xiphidiopsis*) *gurneyi*, mitochondrial genome, phylogeny

## Abstract

*Xiphidiopsis* (*Xiphidiopsis*) *gurneyi* belongs to Meconematinae. The complete mitochondrial genome of *X.* (*X.*) *gurneyi* was sequenced by the next-generation sequencing (NGS) technology. The total length of the mitogenome was 16,225 bp and contains the typical gene arrangement, base composition, codon usage found in Meconematinae species. Phylogenetic tree was constructed based on concatenated datasets of PCGs and rRNAs of *X.* (*X.*) *gurneyi* and 19 Tettigoniidae species to assess their phylogenic relationship. Phylogenetic analysis showed that *X.* (*X.*) *gurneyi* was more closely related to the genus of *Xizicus*.

*Xiphidiopsis* (*Xiphidiopsis*) *gurneyi* is a generic assignment with controversy species, which was reported by Tinkham (1944) within the genus *Xiphidiopsis* (Gorochov et al. 2005; Xiao et al. 2016; Cigliano et al. 2018). Liu et al. (2010) transferred *X.* (*X.*) *gurneyi* to *Euxiphidiopsis* mainly based on the characteristic of vertex disc with two longitudinal bands and the specialized subgenital plate of female. The specimen of *X.* (*X.*) *gurneyi* was collected at Jianfengling in Hainan (18°44′36.91″N, 108°50′34.20″E), China in June 2017 and deposited in Xi’an Botanical Garden of Shaanxi Province. The complete mitochondrial genome of *X.* (*X.*) *gurneyi* was sequenced using the Illumina Hiseq 2500 sequencing platform. Raw reads were filtered to obtain high-quality clean reads by using CLC Genomics Workbench 8 (CLC Bio, Aarhus, Denmark) and then aligned to the mitogenome of *Xizicus fascipes* (JQ326212) as a reference using MITObim v1.8 (Hahn et al. 2013) and Mira 4.0.2 (Chevreux et al. 2004) to assembly. The whole sequence was annotated using the software Geneious v 10.1.2 (Biomatters Ltd., Auckland, New Zealand) by comparing with the mitogenome of *X. fascipes*. The tRNA genes were predicted using online software MITOS (Bernt et al. 2013).

The complete mitogenome of *X.* (*X.*) *gurneyi* was 16,225 bp in length and had been deposited in GenBank under accession no. MH198673. It contained a typical gene content found in metazoan mitogenomes: 13 protein-coding genes (PCGs), 22 transfer RNA (tRNA) genes, two ribosomal (rRNA) genes, and one control region. Gene order and arrangement were identical to the *X. fascipes* mitogenome. The overall base composition of the whole mitochondrial genome was 36.2% A, 31.3% T, 21.3% C, and 11.2% G, exhibiting obvious anti-G and AT bias (67.5%). The initiation codons of all PCGs were typical with ATN (COII, ATP6, COIII, ND4, ND4L, and Cytb with ATG, ND2, COI, ATP8, ND3, ND5 with ATT; ND6, ND1 with ATA). The ATN codon was prevalently regarded as initiation codons for mitogenome PCGs in insects. Seven genes (ND2, COI, ATP8, ATP6, ND3, ND4L, and ND6) used TAA as the termination codons, and two genes (Cytb, ND1) were stopped with TAG. COII, COIII, ND5, and ND4 had an incomplete termination codon T. The length of tRNA genes ranged from 63 to 71 bp. All tRNA genes had the typical cloverleaf secondary structures except for tRNA^Ser(AGN)^. The lrRNA and srRNA were 1303 bp and 785 bp in length, respectively. The control region was 1448 bp in length and was composed of 58.9% A and T nucleotides.

Phylogenetic analysis was performed using MrBayes 3.1.2 (Ronquist and Huelsenbeck 2003) under the GTR + I+G model chosen by jModeltest (Posada 2008) based on the concatenated datasets of PCGs and rRNAs of 19 Tettigoniidae species which were downloaded from GenBank, with *Phryganogryllacris xiai* and *Tarragoilus diuturnus* selected as the outgroups. The phylogenetic analysis results showed that *X*. (*X*.) *gurneyi* was closely related to the genus *Xizicus* (Figure 1), which was congruent with the phylogenetic results based on combined dataset of COI and ITS1-5.8S rDNA-ITS2 (Han 2016).

**Figure 1.**
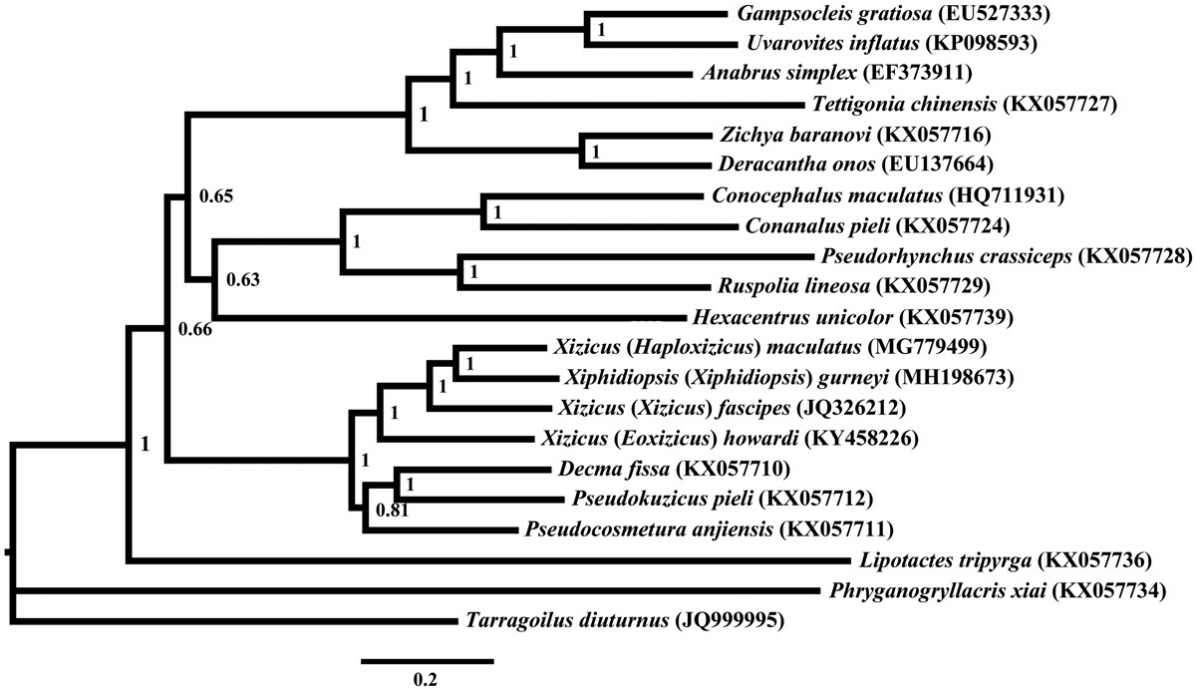
Phylogenetic reconstruction of Tettigoniidae using mitochondrial PCGs and rRNA concatenated dataset.
